# First-in-human trial of Stabilizer device in neuroendovascular therapy

**DOI:** 10.1016/j.heliyon.2023.e14360

**Published:** 2023-03-11

**Authors:** Chiaki Sakai, Nobuyuki Sakai, Ariel Takayanagi, Hirotoshi Imamura, Tsuyoshi Ohta, Masaomi Koyanagi, Masanori Goto, Ryu Fukumitsu, Tadashi Sunohara, Nobuyuki Fukui, Shirabe Matsumoto, Tomoaki Akiyama, Yuki Takano, Hironori Haruyama, Koichi Go, Shinji Kajiura, Masashi Shigeyasu, Kento Asakura, Ryo Horii, Yuji Naramoto, Rikuo Nishii, Yasuhiro Yamamoto, Kunimasa Teranishi, Satohiro Kawade, Taichiro Imahori, Naoki Kaneko, Satoshi Tateshima

**Affiliations:** a Center for Clinical Research and Innovation, Kobe City Medical Center General Hospital, Kobe, Japan; b Department of Neurosurgery, Kobe City Medical Center General Hospital, Kobe, Japan; c Division of Interventional Neuroradiology, Ronald Reagan UCLA Medical Center, Los Angeles, CA, USA; d Department of Neurological Surgery, Riverside University Health System, Moreno Valley, CA, USA

**Keywords:** Clinical trial, First-in-human, Device navigation, Exchange method, Anchor technique, Stent

## Abstract

**Objectives:**

Flow diverter or stent implantation to intracranial target lesion requires large inner diameter microcatheter navigation. The exchange method using stiff long wire is often necessary if it is difficult to navigate over the regular guidewire. However, this method has an intrinsic risk of vessel damage and may cause severe complications. We investigated the safety and efficacy of a new device, the Stabilizer device for navigation in a first-in-human clinical trial under the Certified Review Board agreement.

**Materials and methods:**

The Stabilizer is a 320 cm length exchange wire with a stent for anchoring and is compatible with a 0.0165” microcatheter. The trial design is a prospective single-arm open-label registry. Inclusion criteria are elective flow diverter treatment or stent-assisted coiling, expected to be difficult to navigate a microcatheter with a regular micro guidewire, and obtained documented consent. The primary endpoint of the study was a hemorrhagic complication.

**Results:**

Five patients were enrolled in this trial. The median age is 52 years, ranges from 41 to 70, and all patients were female. Three aneurysms were located on the internal carotid artery, one on the vertebral artery, and one on the basilar artery. Basilar artery aneurysm was treated by stent-assisted coiling and others were treated by flow diverter deployment. All cases successfully navigate microcatheter for the treatment by the trial method using Stabilizer device without any adverse event.

**Conclusions:**

The results from this first-in-human consecutive five cases show the safety of the Stabilizer device in neuro-endovascular therapy for navigation of devices to the intracranial target lesion.

## 1 Introduction

Navigation to the target lesion is the first step in successful neuroendovascular treatment. A larger microcatheter is necessary to deliver the treatment device such as flow diverters. An exchange maneuver is often necessary to navigate a larger microcatheter, especially in challenging tortuous anatomy. A soft, flexible guidewire is replaced with a stiff, supportive exchange-length wire, while a small microcatheter is replaced with a larger microcatheter that will be used for device delivery. Although this exchange maneuver is a useful technique, an accidental forward movement during the exchange can cause vessel injury such as vessel dissection or perforation, while a backward movement can lead to loss of access to the lesion. In the Stenting versus Aggressive Medical Therapy for Intracranial Stenosis (SAMMPRIS) trial in which an exchange maneuver was necessary to deliver the Wingspan stent, vessel perforation occurred in 1.7% of patients [1]. A less precarious method for device exchange could improve the safety of many endovascular procedures.

The Stabilizer is a 320 cm long exchange wire with a retrievable stent designed to assist with exchange maneuvers and device delivery. The stent can keep the wire in a steady position while the microcatheters are being exchanged, with a potential reduction in complications such as vessel dissection, vasospasm, and perforation. A single arm, prospective, open label trial was performed to investigate the safety and efficacy of the Stabilizer. We present the first-in-human series using the device.

## 2 Materials and methods

### 2.1 Study design, patient selection, and data collection

The clinical trial was approved by the Certified Review Board agreement, registered on October 4, 2021 (https://jrct.niph.jgo.jp/en-latest-detail/jRCTs052210098). A single-arm, open-label, non-randomized, prospective, investigator-initiated clinical trial was performed.

#### 2.1.1 Patient enrollment

Inclusion criteria were 1. Scheduled flow diverter treatment or stent assistant coiling, 2. The expectation of difficult navigation using a traditional microcatheter and guidewire. Written consent was obtained from all participants after the risks, benefits, and alternatives of trial participation were explained.

### 2.2 Endpoints

The primary endpoint of the study was a hemorrhagic complication. Secondary endpoints for safety included ischemic stroke, arterial dissection, vasospasm, and any adverse events. The secondary endpoint for efficacy was successful device navigation.

#### 2.2.1 Device components

**Fig. 1:**
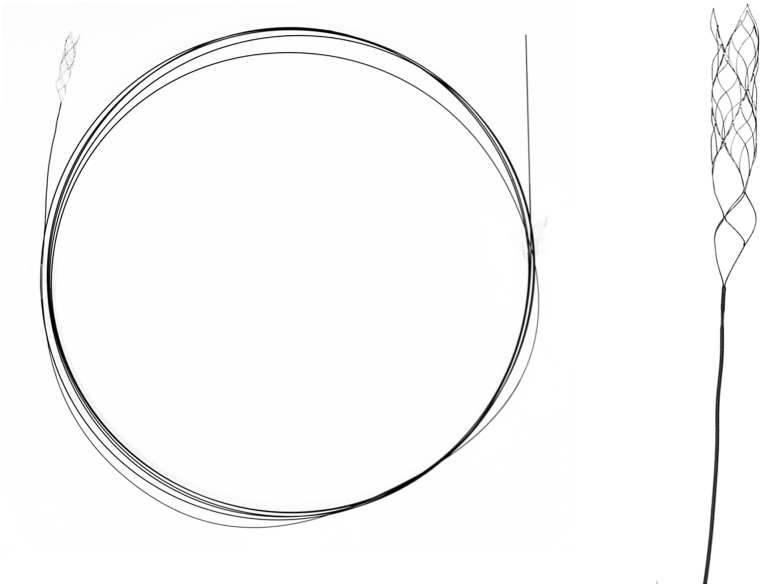
Stabilizer device has a 320 cm exchange-length wire (left) with a stent at the distal end (right), compatible with 0.0165″ microcatheter.

The Stabilizer Device is made by Bolt Medical, Tokyo, Japan (Fig. 1). The device has a self-expanding retrievable stent mounted on the distal end of a 320 cm exchange-length microguidewire. It is compatible with microcatheters that have a 0.0165” inner diameter.

### 2.3 Treatment protocol

A traditional microcatheter and micro guidewire were used to access the target vessel. The micro guidewire was removed and replaced with the Stabilizer which was deployed in the target artery. The microcatheter was then removed and replaced with the larger microcatheter that would be used for treatment. The Stabilizer was then retrieved after resheathing with the microcatheter.

## 3 Results

### 3.1 Baseline characteristics

Five patients were enrolled between October 25 and December 15 in 2021. The median age was 52 years (range 41–70). All patients were female. Three of the aneurysms were located in the anterior circulation and two in the posterior circulation. Specific aneurysm location and size are shown in Table 1.

**Table 1:** Demographics and baseline characteristics.

Case #	Age (years)	Gender	Aneurysm location	Aneurysm size (max, mm)
1	41	Female	R paraclinoid ICA	14 mm
2	52	Female	L paraclinoid ICA	12 mm
3	72	Female	L VA	7 mm
4	66	Female	Basilar artery (trunk)	8 mm
5	56	Female	L cavernous ICA	15 mm

### 3.2 Endovascular procedure and outcome

The basilar artery aneurysm was treated with stent assisted coiling, two patients with flow diversion and coils and two with flow diverters alone. Operative details and outcome are shown in Table 2. In all cases, successful navigation was achieved using the Stabilizer. Asymptomatic vasospasm occurred in one patient during use of the Stabilizer. No other device-related adverse events occurred. One patient had a transient ischemic attack (TIA) after treatment which was unrelated to the device.

**Table 2:** Operative details and outcome.

Case #	Stabilizer dimensions	Location of Stabilizer deployment/segment	Navigation device	Treatment modality	Technical success (Y/N)	Adverse event (device-related)	Adverse event (unrelated to device)
1	4 mm × 15 mm	R MCA/M1	Phenom27	Flow Diverter + coiling	Y	N	N
2	4 mm × 15 mm	L MCA/M2	Phenom27	Flow Diverter + coiling	Y	Asx vasospasm	TIA
3	4 mm × 15 mm	BA	Headway27	Flow Diverter	Y	N	N
4	4 mm × 15 mm	L PCA/P2	Prowler Select Plus	Stent assisted coiling	Y	N	N
5	4 mm × 15 mm	L MCA/M1	AXS Catalyst 5	Flow Diverter	Y	N	N

BA, basilar artery; Asx: asymptomatic, ICA, internal carotid artery; VA, vertebral artery; PCA, posterior cerebral artery; MCA, middle cerebral artery.

**Fig. 2:**
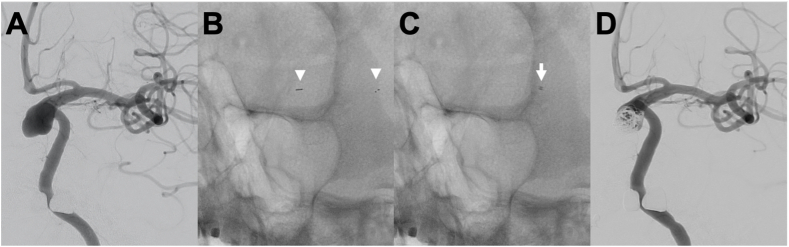
Representative case (Case 2 in Table 1, Table 2), (A) Left carotid artery injection, anteroposterior projection showing a large left paraclinoid ICA aneurysm. (B) Stabilizer unsheathed in the M1 and M2 segment of the left MCA. The two stent markers are indicated by the white arrowheads. (C) After microcatheter exchange over the Stabilizer, the Phenom 27 (distal marker indicated by the white arrow) is positioned in the left M2. (D) Final left ICA injection showing decreased filling of the aneurysm after placing a PED and coiling.

Representative case (Case 2 in Table 1, Table 2, Fig. 2).

A left paraclinoid internal carotid artery (ICA) aneurysm was incidentally found on MRI. Bilateral transfemoral access was obtained and the patient was heparinized, with a right 8 Fr long sheath for flow diverter deployment and a left 6 Fr long sheath for aneurysmal coiling. Through the right femoral access, an 8 Fr FUBUKI guide catheter (Asahi Intecc, Nagoya, Japan) and a 6 Fr JB2 and RFGW 0.035″ were placed in the right common carotid artery (CCA). Through the left femoral artery access, a 6 Fr Envoy catheter with a 4.2 Fr OK2M (Katecs, Nagoya, Japan) and Radifocus Glidewire 0.035” (Terumo, Tokyo, Japan) was used to access the right ICA. An SL-10 (Stryker Neurovascular, Fremont, CA, USA) was navigated into the left M2 segment of the middle cerebral artery (MCA) over a Venture-shaped J (Mizuho, Tokyo, Japan). A Stabilizer 4.5 × 15 mm was then deployed through the SL-10 in the distal M1 and the SL-10 was exchanged for an AXS Catalyst 5/Offset (Stryker) for the deployment of a Surpass Streamline (Stryker). During the exchange, the tip of the Stabilizer did move to the mid M1 segment, but access was maintained. The Stabilizer wire became looped so it was pulled back and AXS Catalyst 5 was advanced to the M1. When a right ICA angiogram was obtained, a spasm beyond the ICA terminus with contrast stagnation was seen. Therefore, the AXS Catalyst 5 was then pulled back to the C2 segment of the ICA without improvement but once it was pulled further back to the CCA the vasospasm resolved. The SL-10/Venture was navigated from the Fubuki to the M2 segment and the Stabilizer was again deployed in the distal M2 (Fig. 2B). The SL-10 was then exchanged for a Navien 115 cm (Medtronic Neurovascular, Irvine, CA, USA) and Phenom 27 150 cm (Medtronic) for the deployment of a Pipeline Flex (Medtronic). There was no change in the position of the Stabilizer during the exchange, but the delivery wire again became looped. The Stabilizer was again pulled back and the Phenom 27/Navien were advanced to M2 and the Stabilizer was removed (Fig. 2C). A control angiogram from the right CCA showed good flow through the anterior cerebral artery (ACA) and MCA. The SL-10 was used to access the aneurysm through the ENVOY prior to deploying the Pipeline Flex 4.25 × 16 mm (Medtronic) from the M1 to the distal ICA. With the SL-10 jailed in the aneurysm, 3 Target XL (Stryker) coils were placed in the aneurysm and the SL-10 was removed. A Transform 7 × 7 mm (Stryker)/Chikai 14 (Asahi Intecc) was used to obtain better apposition of the Pipeline flex to the vessel wall. A final angiogram was performed along with VasoCT which showed good wall apposition (Fig. 2D). The patient recovered well post-operatively and remained without neurological deficits.

## 4 Discussion

During endovascular treatment, an exchange maneuver is used to replace the navigable catheters to the larger, more cumbersome device delivery catheters. Exchange maneuvers have been reported to have a vessel perforation rate as high as 3% and can lead to mortality in 1.3–4.6% of patients who are on dual antiplatelet therapy [2]. The Stabilizer, a 320 cm exchange wire with a self-expanding stent at the distal end (Fig. 1) is designed to assist with exchange maneuvers and prevent unintended movements of the exchange wire that can lead to vessel injury or perforation. In this case series, we describe the first in-person experience using the Stabilizer. Successful navigation was achieved in all five cases. One patient had asymptomatic vasospasm during the use of the Stabilizer but no other device-related events occurred. One patient had a TIA, which was adjudicated as an unrelated event to the study device.

Several techniques have been proposed to maintain access during microcatheter exchanges including the use of balloons [[3], [4], [5]], stents [6,7], stent retrievers [8,9], and microwires [10]. A balloon can be used in a similar fashion to the Stabilizer to prevent accidental movement of the guidewire. However, the Stabilizer has the added advantage of allowing blood flow through the vessel during the exchange [[8], [9], [10], [11]]. Traditional stent retrievers have also been used for difficult navigation or exchange maneuvers but because the push wire is not long enough, special precautions are necessary to prevent air embolism [9]. Additionally, the operator does not have control of the wire while the new catheter is being advanced. The Stabilizer, however, allows the operator to be in control of the wire throughout the exchange and does not require extra steps to prevent air embolism compared to a traditional exchange wire.

A recent in vitro study demonstrated significantly greater success with the Stabilizer when navigating challenging settings and performing exchange maneuvers with stiff devices compared to a traditional guidewire [11]. In this first-in-human experience using the Stabilizer, we were able to successfully navigate the device in all five cases with no clinically significant adverse events. Although vasospasm occurred in one patient, it resolved without treatment and the patient was asymptomatic.

The Stabilizer has the potential to be used in other procedures, such as in the treatment of intracranial stenosis. The Stabilizer, by stabilizing the position of the distal end of the wire during the exchange and advancement of the stiff stent, has the potential to prevent accidental movements and vessel perforation during the procedure. The Stabilizer also may be used during the treatment of challenging giant aneurysms by maintaining access during the straightening of redundant loops of a catheter.

One limitation of this study is the small sample size. Future prospective studies will be necessary to determine the safety and efficacy of the device.

## 5 Conclusion

In this first-in-human trial, we demonstrated procedural success in all five patients when using the Stabilizer for exchange maneuvers. On patient had angiographic vasospasm but no clinically significant device-related events occurred.

## Author contribution statement

Chiaki Sakai, Nobuyuki Sakai, Ariel Takayanagi, Hirotoshi Imamura, Tsuyoshi Ohta, Masaomi Koyanagi, Masanori Goto, Ryu Fukumitsu, Tadashi Sunohara, Nobuyuki Fukui, Shirabe Matsumoto, Tomoaki Akiyama, Yuki Takano, Hironori Haruyama, Koichi Go, Shinji Kajiura, Masashi Shigeyasu, Kento Asakura, Ryo Horii, Yuji Naramoto, Rikuo Nishii, Yasuhiro Yamamoto, Kunimasa Teranishi, Satohiro Kawade, Taichiro Imahori, Naoki Kaneko, Satoshi Tateshima: Conceived and designed the experiments; Performed the experiments; Analyzed and interpreted the data; Contributed reagents, materials, analysis tools or data; Wrote the paper.

## Funding statement

This research did not receive any specific grant from funding agencies in the public, commercial, or not-for-profit sectors.

## Data availability statement

Data included in article/supp. material/referenced in article.

## Declaration of interest's statement

The authors declare the following conflict of interests: This trial received trial devices from Bolt Medical, without specific grant. Grant from Kobe City Medical Center General Hospital support this trial. The authors, NS, NK and ST, were consultants of Bolt Medical at the treatment, but declare that this trial was conducted in the absence of any commercial or financial relationships that could be constructed as a potential conflict of interest. 10.13039/100007057NS received a research grant from Biomedical Solutions, 10.13039/501100002973Daiichi-Sankyo, and Terumo; lecturer's fees from Asahi-Intec, Biomedical Solutions, 10.13039/501100002973Daiichi-Sankyo, and Medtronic; membership on the advisory boards for Johnson&Johnson, 10.13039/100004374Medtronic and 10.13039/501100008645Terumo without related this manuscript. HI received lecturer's fee from 10.13039/100004374Medtronic. NK has been a consultant for Stryker and Medtronic. 10.13039/501100004347ST received research funds from Biomedical Solutions, Rapid Medical and Medtronic, and a consultant for TG Medical, 10.13039/100008476Irvine Neurovascular, Balt USA, Cerenovus, 10.13039/100004374Medtronic, Phenox GmbH, MicroVention, Kaneka USA, Century Medical Inc., EnCompass, NVMedTech, and 10.13039/100008894Stryker. The other authors have no personal or financial interest in any of the materials or devices described in this article.
